# Low-dose aspirin as a promising agent for the prevention of spontaneous preterm birth

**DOI:** 10.1136/ebnurs-2018-102998

**Published:** 2019-05-30

**Authors:** Anadeijda J E M C Landman, Martijn A Oudijk

**Affiliations:** 1 Department of Obstetrics & Gynaecology, Amsterdam UMC, Vrije Universiteit Amsterdam, Amsterdam Reproduction & Development, de Boelelaan 1117, Amsterdam, the Netherlands; 2 Department of Obstetrics & Gynaecology, Amsterdam UMC, University of Amsterdam, Amsterdam Reproduction & Development, Meibergdreef 9, Amsterdam, the Netherlands

**Keywords:** perinatology, preventive medicine, obstetrics

**Commentary on**: Andrikopoulou M, Purisch SE, Handal-Orefice R, *et al*. Low-dose aspirin is associated with reduced spontaneous preterm birth in nulliparous women. *Am J Obstet Gynecol*. 2018;219:399.e1–399.e6.

## Implications for practice and research

- At this moment, there is insufficient evidence to implement low-dose aspirin for the prevention of spontaneous preterm birth.
- Placebo-controlled randomised controlled trials are needed to confirm the effectiveness of aspirin for the prevention of spontaneous preterm birth.

## Context

Preterm birth is the most important cause of perinatal morbidity and mortality worldwide. Despite the administration of progesterone to women with a previous spontaneous preterm birth, ~20%–30% will deliver preterm again.^1^ Accumulating evidence suggests that uteroplacental ischaemia plays an important role in the aetiology of spontaneous preterm birth, comparable to its role in preeclampsia. As aspirin is proven to reduce the risk of developing preeclampsia, it is hypothesised that aspirin might also be effective to prevent spontaneous preterm birth through its antithrombotic and anti-inflammatory effect. Several studies have shown promising results.^2–4^

## Methods

The objective of the study by Andrikopoulou *et al* 4 was to evaluate the effect of low-dose aspirin for the prevention of spontaneous preterm birth in nulliparous women without comorbidities. All were singleton and gestations without congenital malformations. This was a secondary analysis of a placebo-controlled randomised controlled trial preformed from 1989 to 1991 with preeclampsia as primary outcome.^5^ Treatment with aspirin or placebo was initiated between 13 and 25 weeks of gestation. The primary outcome of the present study was spontaneous preterm birth <34 weeks of gestation.

## Findings

A total of 2543 women were included of which 1262 (49.6%) received 60 mg aspirin and 1281 (50.4%) placebo. Spontaneous preterm birth <34 weeks occurred significantly less in the aspirin-group (1.03%) compared with the placebo-group (2.34%); RR 0.46 (95% CI 0.23 to 0.89). This effect was independent of time of initiation of therapy (<16 vs ≥16 weeks) and was also significant after exclusion of women who developed preeclampsia (n=2). There was no difference in spontaneous preterm birth <37 weeks of gestation.

## Commentary

The hypothesis that aspirin might be effective for prevention of spontaneous preterm birth is based on overlapping pathophysiology with preeclampsia. In this respect, two striking elements of the study by Andrikopoulou *et al* should be addressed. First, the parent trial did not show any benefit of low-dose aspirin with regards to preeclampsia.^5^ The incidence of preeclampsia was 6.3% in the aspirin group and 4.6% in the placebo group (RR 0.7; 95% CI 0.6 to 1.0). Second, the median gestational age at randomisation was 20 weeks. Sensitivity analysis demonstrated that gestational age at initiation of therapy did not influence the results with regard to spontaneous preterm birth, while in case of preeclampsia previous studies have shown that it is best to start with aspirin between 8 and 16 weeks of gestation.^6^

Andrikopoulou *et al* suggest that aspirin may either have a different or additional mechanism of action in the prevention of spontaneous preterm birth compared with its use in the prevention of preeclampsia. Initiation of aspirin therapy between 8 and 16 weeks of gestation is important for prevention of preeclampsia because this is the period that placentation and transformation of spiral arteries occurs. We speculate that in case of spontaneous preterm birth the anti-inflammatory properties of aspirin are beneficial throughout pregnancy.

The results of studies performed thus far on the effect of aspirin for prevention of spontaneous preterm birth are promising; however, more placebo-controlled randomised controlled trials are needed to confirm these results and to assess in which subgroups it is effective. To gain more insight in the mechanism of action, we urge research groups to perform translational research alongside healthcare evaluation studies through biosampling: that is, with blood samples and placenta or placental bed biopsies.
